# Inundation and Fire Shape the Structure of Riparian Forests in the Pantanal, Brazil

**DOI:** 10.1371/journal.pone.0156825

**Published:** 2016-06-09

**Authors:** Wellinton de Sá Arruda, Jens Oldeland, Antonio Conceição Paranhos Filho, Arnildo Pott, Nicolay L. Cunha, Iria Hiromi Ishii, Geraldo Alves Damasceno-Junior

**Affiliations:** 1 Laboratório de Botânica, Centro de Ciências Biológicas e da Saúde, Universidade Federal de Mato Grosso do Sul, Campo Grande, MS, Brazil; 2 Biodiversity, Evolution, and Ecology of Plants (BEE) Biocenter Klein Flottbek and Botanical Garden, University of Hamburg, Hamburg, Germany; 3 Laboratório de Geoprocessamento para Aplicações Ambientais, Faculdade de Engenharias, Arquitetura e Urbanismo e Geografia, Universidade Federal de Mato Grosso do Sul, Campo Grande, MS, Brazil; 4 Programa de Pós-Graduação em Ecologia e Conservação, Universidade Federal de Mato Grosso do Sul, Campo Grande, MS, Brazil; 5 Laboratório de Botânica, Campus do Pantanal, Universidade Federal de Mato Grosso do Sul, Corumbá, MS, Brazil; Wuhan Botanical Garden,CAS, CHINA

## Abstract

Inundation and fire can affect the structure of riparian vegetation in wetlands. Our aim was to verify if there are differences in richness, abundance, basal area, composition and topographic preference of woody species in riparian forests related to the fire history, flooding duration, or the interaction between both. The study was conducted in the riparian forests of the Paraguay River some of which were burned three times between 2001 and 2011. We sampled trees with a girth of at least 5 cm at breast height in 150 5 × 10 m plots (79 burned and 71 unburned). We also measured height of the flood mark and estimated the flooding duration of each plot. We performed Generalized Linear Mixed Models to verify differences in richness, basal area, and abundance of individuals associated to interaction of fire and inundation. We used an analysis of similarity (ANOSIM) and indicator species analysis to identify differences in composition of species and the association with burned and unburned area according to different levels of inundation. Finally, we used a hierarchical set of Generalized Linear Models (GLM), the so-called HOF models, to analyse each species’ specific response to inundation based on topography and to determine their preferred optimal topographic position for both burned as well as unburned areas. Richness was positively associated with elevation only in burned areas while abundance was negatively influenced by inundation only in burned areas. Basal area was negatively associated with time of inundation independent of fire history. There were 15 species which were significant indicators for at least one combination of the studied factors. We found nine species in burned areas and 15 in unburned areas, with response curves in HOF models along the inundation gradient. From these, five species shifted their optimal position along the inundation gradient in burned areas. The interaction of fire and inundation did not appear to affect the basal area, but it did affect the richness, number of individuals, success of some species, and seemed to shape the boundary of these forests as shown by the difference in the positioning of these species along the inundation gradient.

## Introduction

Fire in wetlands is an interesting phenomenon because its occurrence puts into play two opposing extreme events, fire and inundation, both of which have a profound effect in the riparian vegetation. According to the flood pulse concept [1], plant communities of floodplains are unique and need periodical inundation to maintain their composition and structure. The seasonal inundation can promote changes in physiology and morphology of plants such as hypertrophied lenticels and anaerobic metabolism [2, 3]. At the landscape level, inundation is associated with changes in distribution of communities, species zonation in riparian forests, as well as richness and density of trees [1, 4–6]. Inundation can also result in reduction of growth rates, biomass production, basal area, and increased resprouting ability [7,8]. Furthermore, it affects the recruitment efficiency of plants in the seedling phase [9] and interacts actively in seed dispersal [10].

In subtropical and tropical seasonally flooded landscapes, such as the Okavango delta, the Everglades, or the Brazilian Pantanal, riparian forests are also subjected to regular fire events [11–16]. Generally, not all fires are lethal for most plant species. Some species have physical protection against fire, such as thick bark or a specific trunk profile [17, 18], while others have evolved with physiological advantages which resist fire such as fast growth [17], the ability to protect the photosynthetic apparatus from fire [19], and ability to resprout or even germinate better after a fire [20]. The effects of fire in riparian forests are still poorly understood. In systems with neighboring seasonally flooded savannas, the presence of grasses plays an important role in fire events. Fires tend to start in grassy vegetation and then move to the forests located in higher areas [21]. Its intensity varies according to the type of fuel from arboreal and herbaceous components [22]. Grasses, when burned, can promote the top kill of small trees and, as a consequence, these fire events act as a filter for woody species recruitment [23, 24]. Depending on the intensity of inundation (duration and speed of water), fire may have a stronger effect in some areas and eliminate trees surrounded by dead trunks brought by high waters [25, 26]. In general, depending on frequency, intensity, extent, season, and rate of spread, fire can promote changes in phenology, species composition, biomass, structure, resprouting, and productivity of riparian environments [27, 28].

Many species occur in riparian landscapes according to their physiological ability to adapt or resist effects of inundation [3, 29] or to the ecology of dispersal [10, 30]. As fire can eliminate trees adjacent to grasslands, frequent fires may interact with inundation effects, changing the microtopographic optimum of occurrence of species along the flood gradient. Despite fire not being as predictable as inundation, it is possible that fire can act together with inundation as an important factor in structuration of riparian forests. In this context, it is important to investigate the variation of the structure of these forests in the presence of recurring fire events and the combination of fire and inundation which influence structural variation in riparian forests. Such information could help in the management and conservation policies of these riparian forests.

The Pantanal is a Cenozoic floodplain of more than 140,000 km², originated from the sinking of the high Paraguay River basin [31], and located in central South America, mainly in Brazil with some extensions into Paraguay and Bolivia [32]. The Paraguay River is the main river and has many tributaries, with riparian forests subjected to seasonal inundation which can occur for more than 200 days a year [5]. In the last 14 years, fires have been frequent on its floodplain with at least 4 big events observed in field. The interaction between fire and inundation can have an influence on richness and abundance of the regrowth of these riparian forests [33]. However, the effects of the interaction between fire and inundation on the structure are poorly understood for floodplains in general [27] and have not been studied yet for adult trees in these riparian forests.

In this study, we examine how the interaction of inundation and fire is associated with differences in the structure of the adult trees of riparian forests along the Paraguay River in burned and unburned areas. To achieve this goal, we addressed the following questions: (1) Do inundation, fire history, including their interaction, affect the aspects of basal area, tree abundance and richness? We predicted that higher values of basal area, richness and number of individuals, i.e. abundance, would be more likely to occur in areas that suffer minor effects of inundation. Moreover, we would expect that periodical fires interacting with inundation would cause reduction in number of individuals, number of species, and loss of biomass. Lower biomass would be also associated with a lower investment in growth in areas having higher levels and time of inundation, consequently reducing basal area. (2) Are there differences in the species composition and/or indicator species related to the interaction of inundation and fire? We predicted changes in the composition of species in burned areas and the existence of species that can be considered as indicator species for the different combinations of fire occurrence and levels of inundation. (3) Does fire lead to changes in the positioning and species specific responses across the inundation gradient? Fire can decrease abundance, especially in lower areas at the transition to floodable grasslands where fire is more intense. Thus we predicted that for some species, fire would lead to a shift of the specific response and optimal position along the flood gradient to higher areas compared to the unburned areas for that same species.

## Materials and Methods

### Study area

**Fig 1:**
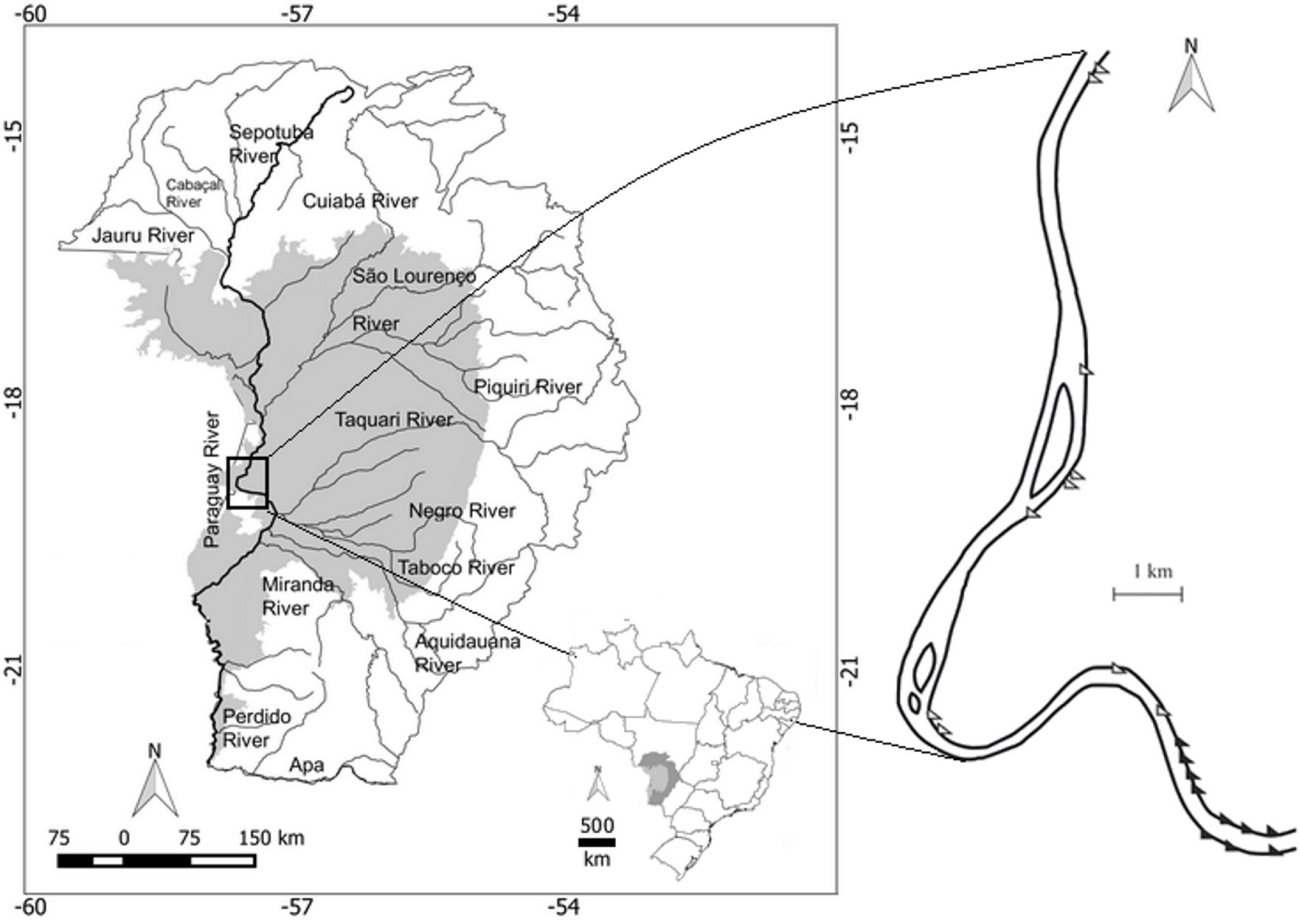
Study area showing the Brazilian Pantanal (gray) with the headwaters (white) and the transect areas along the Paraguay River. Black triangles indicate unburned areas, and white triangles indicated areas burned in 2001, 2005, and 2009.

This study was conducted in the riparian forests along the Paraguay River between 18°51'7.26" S, 57°36'50.20" W and 18°58'27.39" S, 57°38'8.41" W upstream from the town of Corumbá in the Pantanal of the Paraguay sub-region, Mato Grosso do Sul State, Brazil (Fig 1). The authorization to work in the areas was provided by INCRA (National Institute of Agrarian Reform).

The topography of the floodplain is flat with slopes ≤ 2 cm/km north-south. The river has numerous secondary channels, meanders, oxbow lakes and islands. The riparian forests are located mainly in levees, interspersed among floodable grasslands. The riparian forests are classified as “seasonal alluvial semideciduous forest” by the Brazilian system of vegetation classification [34] and are dominated by *Inga vera*, *Triplaris gardneriana*, *Ocotea diospyrifolia* and *Crataeva tapia* [5].

**Fig 2:**
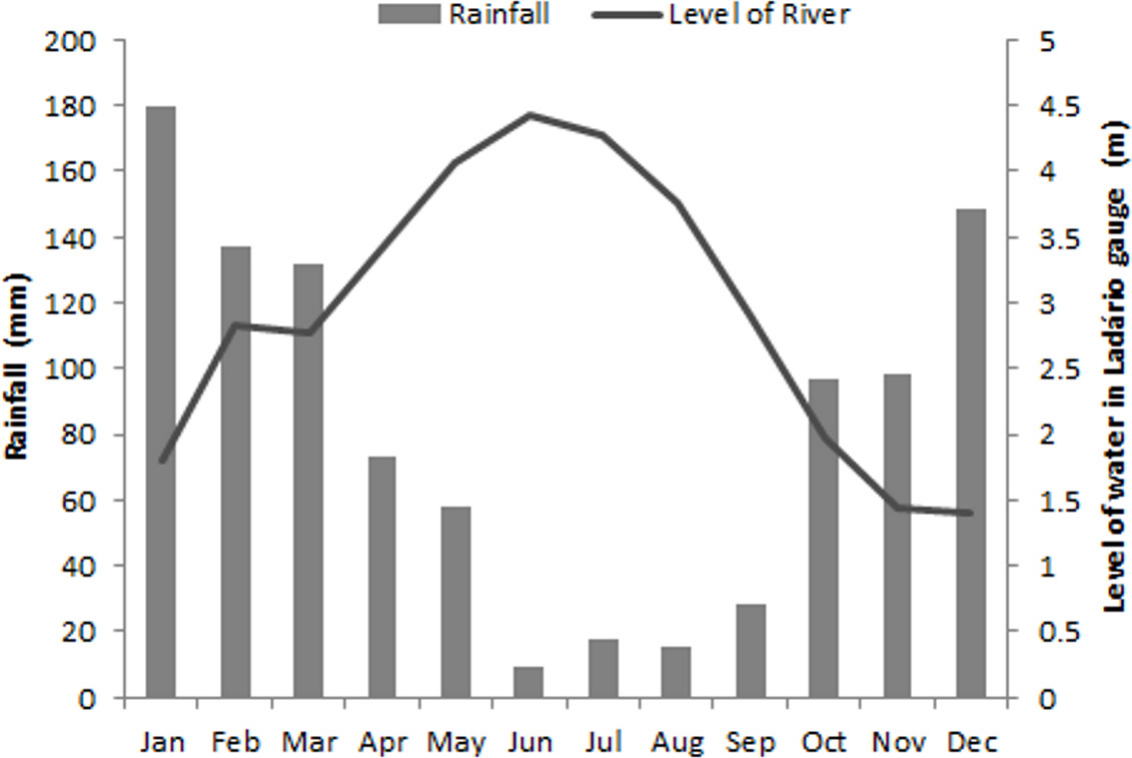
Levels of inundation of the Paraguay River between 2000 and 2013 (m) and monthly mean of rain (cm) in the municipalities of Corumbá and Ladário, MS, Brazil. Data provided by Brazilian Navy and INMET (Brazilian National Institute of Meteorology).

According to the Köppen-Geiger classification [35], the climate is classified as “Awa” with a distinct wet (summer) and dry (winter) season, with mean annual rainfall of 1070 mm [36]. The flooding regime of the Pantanal is variable according to the sub-region and the overflowing river. In the Paraguay River it is seasonal, unimodal, and predictable. The inundation wave begins to rise in December/January, peaks in May/June at the middle of dry season, and ends in December during the rainy season (Fig 2). This delay of the wave occurs because it takes about three months to arrive from the headwaters to Corumbá. The flooded area in the floodplain of Paraguay River, which is the lowest part of the Pantanal, varies from 4,000 to 16,000 km^2^ between low and high water seasons [37]. In a broad sense, the Paraguay River overflows when its level reaches over 4 m at the Brazilian Navy Ladário Gauge [38]. The riparian forests remain inundated from 11 to 220 days per year, depending on the maximum attained by the river, the height of the riverbank, and the position of each tree on the levee [5].

The fire regime is determined by a combination of the inundation regime and the occurrence of rain. As most places are inundated during the dry season, fire occurs mainly at the end of the dry season in years when the peak of inundation does not attain 4 m in the Ladário gauge [37] or when rainfall is below average in December or January. As the floodplain is dominated by fast-growing floodable grasslands [39], a considerable amount of dry biomass serves as fuel. The origin of these fires is not yet well established, certainly natural phenomena such as lightning play an important role, but the main possible causes seem to be accidental and intentional human use as a management tool (G. A. Damasceno-Junior, personal observation).

The prevalent soils in the floodplain are gley soils, typical for floodplains, poorly drained, mainly eutrophic, clayey to moderately clayey. On the riverbank, there is a sandy superficial layer over a clay horizon, which is thicker on the higher parts and thinner towards the flooded grasslands [40].

### Data collection

**Fig 3:**
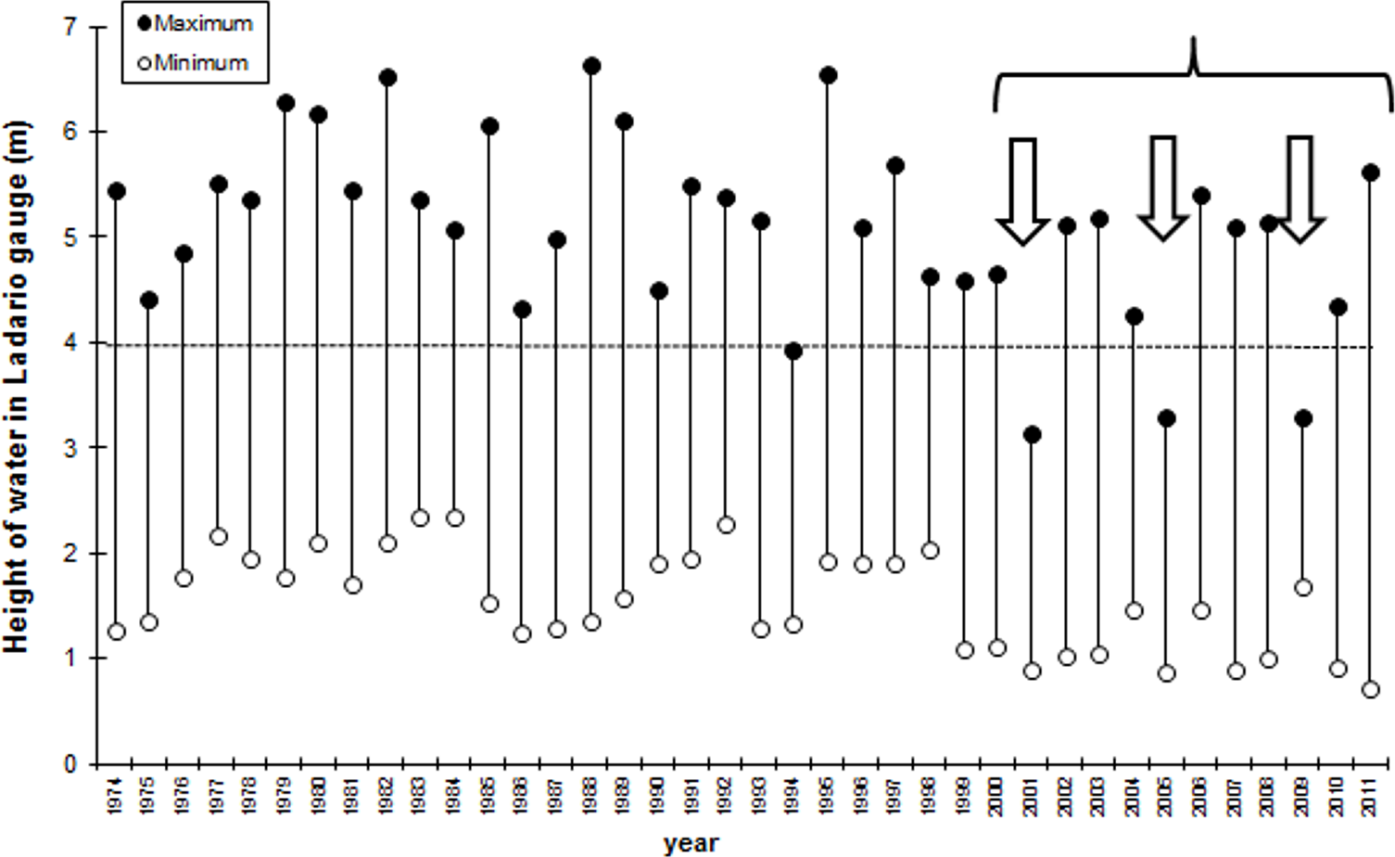
Maximum and minimum levels attained by Paraguay River (m) between 1974 and 2011 in relation to the zero of Ladário gauge, showing the study period (brace) and the years selected as the basis for the fire investigation (arrows). The dashed line indicates a general threshold for inundation of the Paraguay River.

The sampling units were chosen based on the inundation and fire history between 2001 and 2011. We examined Landsat satellite images provided by the Brazilian National Institute of Spatial Research specifically corresponding to the years in which an inundation below 4 m and a dry period extending until October or November were observed (*i*.*e*., 2001, 2005 and 2009; Fig 3). Based on satellite images from these years, we selected twenty study sites in the riparian forests that were either burned (10) or remained unburned (10) during the entire study period. We considered as burned sites those completely burned during three fire events (2001, 2005 and 2009), according to the photointerpretation of the Landsat 5 images in different false color composition, using the combination of bands 752 or 543. In other words, only areas completely burned three times were considered as burned areas.

The field work was performed between September and December of 2011. At each study site, we established one transect of contiguous 10 × 5 m plots, with the line of 5 m always being perpendicular to the river bed (Fig 1). Each transect was established to cover the belt of the riparian forest from the river shore to the borders with the floodable grasslands. The number of contiguous plots in each transect varied according to the width of the riparian forest. In total, we sampled 20 transects with 150 plots being 79 in burned areas and 71 in unburned areas. We took special care to have all transects crossing the major variation in topography of the studied levees, which was of most importance within this study since it can be interpreted as a proxy for inundation intensity and duration (see below). We considered the 50 m² plots as the sampling units in our models nested within each transect.

We sampled all individual trees and shrubs, including palms, with stems or at least one branch with girth of 5 cm or more at breast height (1.3 m above ground level) and above 2.5 m high. The circumferences were measured at 1.3 m high for basal area calculations. The branches in individuals ramified below 1.3 m were also recorded. In these cases Basal areas were calculated separately, and the sum of these measures were accounted as one individual. We considered each individual separately, independent of its number of branches. Voucher specimens were collected, dried, and deposited in the Herbarium CGMS of Universidade Federal de Mato Grosso do Sul.

Each measured individual exhibited a water mark left by the last flooding on the bark. We measured the height of the water mark in relation to the ground. Because of the proximity of the study area from the Ladário gauge and the flat topography (2 cm/km), we were able to convert the water mark measures to topographic positions in meters relative to the gauge (see [5]). This gauge has a fixed zero elevation at 82.15 m asl. We then used the transformed values to estimate the duration of inundation for each plot. The water height values per plot were obtained as the mean of the water mark for the trees inside one plot.

### Regression analysis of structural parameters

The systematic distribution of plots along transects can show some degree of spatial dependency among sampling units and thus produce confounding effects due to topographic gradient. In order to reduce any possible bias within this issue we used transects as a random factor in generalized linear mixed models (GLMM) [41]. To answer our first objective, we tested if the basal area, abundance and richness of woody species were related to the fire regime and to the levels of inundation or their interaction. We modelled the basal area, abundance and richness using a GLMM with appropriate statistical distributions, and used fire, inundation and their interaction as fixed effects. We calculated R² value for the GLMM according to Nakagawa & Schielzeth [42]. Finally, we checked the residual pattern of all models for signs of spatial autocorrelation following Zuur et al. [43], i.e. inspecting omni-directional and N,S,W,E directional semivariograms for significant pattern; none of the models exhibited patterns of spatial autocorrelation.

### Species composition and Indicator Species Analysis

To investigate if fire leads to differences in species composition across the inundation gradient we used two approaches: In order to detect differences between the classes that represented the combinations of the environmental factors, we calculated the analysis of similarity (ANOSIM) [44]. ANOSIM calculates the differences of the ranked dissimilarities between and within *a-priori* specified groups. This method calculates an R value that can be interpreted as the amount of overlap of the groups in multivariate space. R values range from 0 to 1, with values less than 0.5 indicating strong overlap and values of more than 0.75 indicating clearly different clusters in multivariate space.

We also verified whether specific species occur significantly more often in certain habitat combinations (*e*.*g*., burned areas with high inundation). Thus, we combined burned and unburned areas with a high and low inundation factor by dividing the inundation gradient into two parts (*i*.*e*., half the total length of the inundation gradient): the lowest part had 74 plots, being 21 burned and 53 unburned, and the highest part had 76 plots, being 58 burned and 18 unburned. This combination was chosen based on the findings of Damasceno-Junior et al. [5] about the variation in composition of species according to levels and time of inundation. We compared the found range of topographic positions of the riparian forest in relation to Ladário gauge with data of Damasceno-Junior et al. [5]. We found that these topographic positions could be separated into two groups divided nearly in the middle. One group with places that inundate from 30 to 60 days a year and do not flood every year and another group which inundates from 60 to 100 days a year with very predictable inundation (almost every year). We performed an indicator species analysis [45] based on relative abundances of species in order to identify significant associations of species to a grouping factor based on an indicator value (*IndVal*). We used a modified version of the original *IndVal*, the group-equalized *IndVal*_*g*_ introduced by De Cáceres *et al*. [46]. This version allowed us to identify the significance of indicator values for multiple hierarchically structured groups.

For the species found as indicators we searched for fire related functional traits based on field observations and information found in Pott & Pott [11]. This was performed to better understand the main strategies used by these species against fire at the community level.

### Changes in species response across the flood gradient

We were also interested in examining how species respond to the inundation gradient in burned and unburned sites, *i*.*e*., do species show a clear optimum along the inundation gradient or do optima differ for burned and unburned sites? Thus, we generated species response curves [47] based on the hierarchical framework of Huisman, Olf, and Fresco [48], the so-called “HOF models”. HOF models are typically used for quantifying the type of species responses along ecological gradients, e.g. linear, unimodal etc. [49]. This is often interpreted as measuring the ecological niche of a species [50]. In the HOF model framework, seven GLMs with increasing complexity are fitted to the data, *e*.*g*. an intercept model, a linear model, a unimodal model, etc., (see Uğurlu & Oldeland [50] for visualization). We identified the model with the best fit using the AICc (Akaike Information Criterion for small samples [51]), which compares the sum of the Kullback-Leibler distances of the residuals for all models. The model with the smallest AIC is chosen as the best out of the seven candidate models.

In this analysis, the plots and transects were disregarded, and the analyses were based on the numbers of individuals per inundation class in relation to the Ladário gauge zero, calculated based on the water mark of each individual. These inundation classes were calculated in half-meter steps along the inundation gradient for burned and unburned areas separately. For the burned areas subset, we used 27 classes of 5 cm each from 3.7 to 5.1 m, while for the unburned areas we sampled only 16 classes from 3.7 to 4.5 m. We reported only models that showed unimodal responses, i.e. the HOF model type IV (unimodal), V (unimodal left skewed), VI (unimodal right skewed), and VII (multimodal; see Jansen & Oksanen [47]). The models type I (without response), type II and III (without optimum) were ignored since these were uninformative with regard to our question.

All analyses were conducted in R [52] with the packages “lme4” [53] for the calculation of the GLMM model, “MuMIn” [54] for *R*^2^ calculations for the GLMM, “labdsv” [55] and “vegan” [56] for multivariate analysis, “indicspecies” [57] for indicator species analysis, “eHOF” [46] for species response curves, “stargazer” [58] for specific model output and “ggplot2” [59] for graphics. As is not possible to visualize as a graphic the GLMM models, we showed graphics of GLM analysis. We used *glm*.*nb* from MASS [60] and visualization of interactions with visreg [61]. It was done because the results were very similar for both analyses.

## Results

### Inundation measurements

Trees in the surveyed plots displayed water marks at heights ranging from 0.77 to 1.83 m. The maximum level of inundation at the Ladário gauge in 2011 was 5.62 m. Thus, we calculated the relative inundation height as 5.62 m minus the mean water mark per plot in field, resulting in a minimum value of 3.79 m and a maximum of 4.85 m of elevation relative to the Ladário gauge. In the 10 years examined, the plot on the highest topographic position remained inundated for a mean of 38 d/yr (SD: 38; minimum = 0; maximum = 96 d/yr), while the lowest topographic position was inundated for a mean of 98 d/yr (SD: 68; minimum = 0; maximum = 172 d/yr).

### Richness

**Fig 4:**
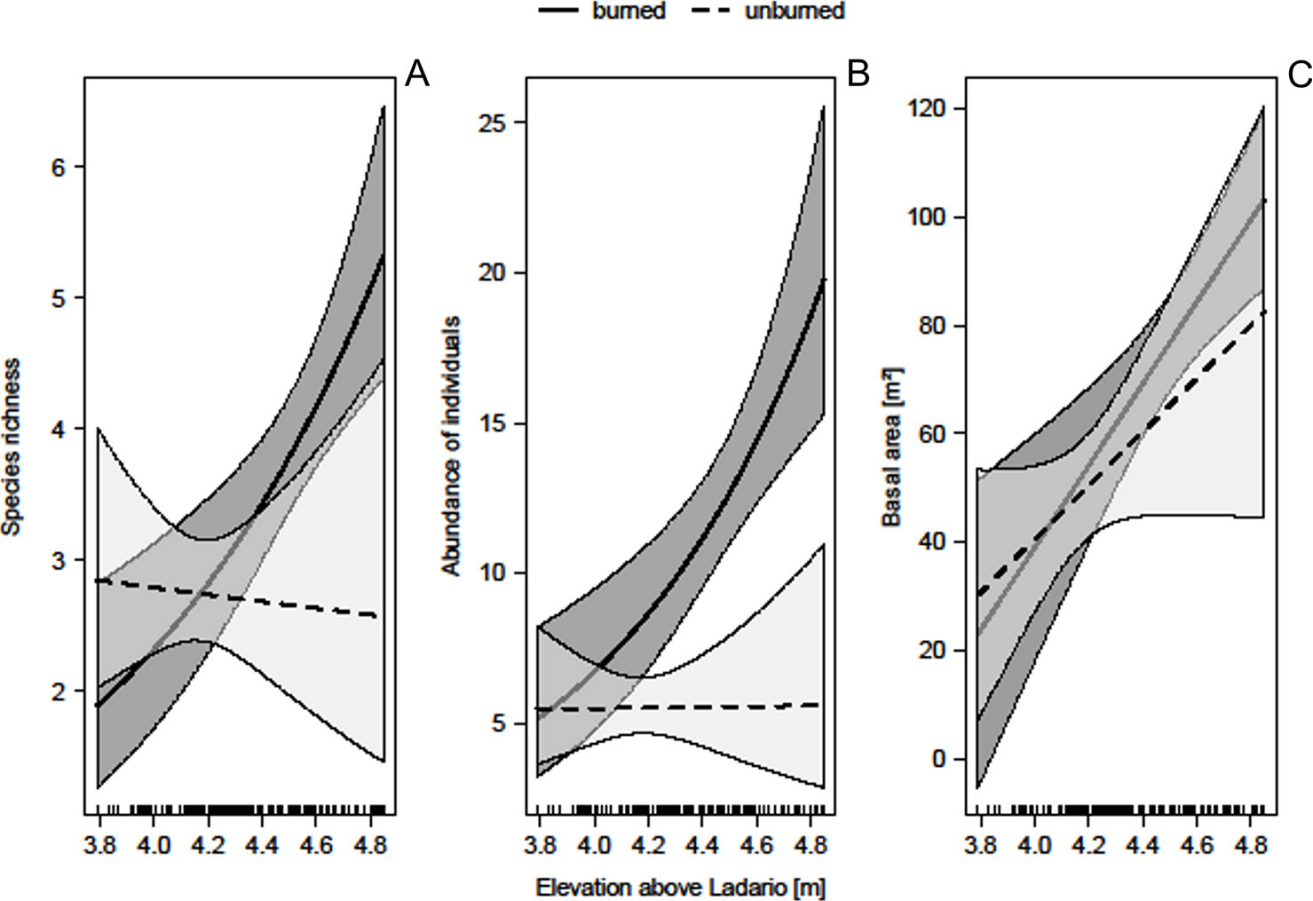
Relationship between the three dependent variables (richness, abundance and basal area) and the interaction of time of inundation and fire occurrence in riparian forests of the Paraguay River. Graphic performed with Generalized Linear Model only for visualization purposes once the results of GLM and GLMM were very similar. The continuous and dotted lines are burned and unburned areas respectively. The shaded areas in both lines are confidence intervals.

We found 39 species from 30 genera and 18 families. The main botanical families were Fabaceae (25.6%), Myrtaceae (18%), Polygonaceae (7.7%), and Euphorbiaceae (7.7%). Of the species, 33.3% were found only in burned areas, 18% only in unburned areas, and 48.7% occurred in both (Table 1). The richness in burned areas was 29 species and in unburned areas 25 species. In the Poisson-GLMM, we found a significant interaction between fire regime and position in the inundation gradient (i.e., time inundated along the year; Table 2). The species richness of shrubs and trees increased with elevation in burned, but not in unburned areas (Fig 4A). In other words, the lower the time of inundation in burned areas, the higher the number of species able to occupy and establish themselves in the flooded habitats.

**Table 1:** List of shrub and tree species found in the riparian forest of the Paraguay River in burned and unburned areas between 2001 and 2011.

Family	Species	Unburned areas (n = 71)	Burned areas (n = 79)
**Apocynaceae**	*Thevetia bicornuta* Müll. Arg.	X	
**Arecaceae**	*Bactris glaucescens* Drude	X	X
	*Bactris major* Jacq.	X	
**Bignoniaceae**	*Handroanthus heptaphyllus* (Vell.) Mattos	X	
**Capparaceae**	*Crataeva tapia* L.		X
**Elaeocarpaceae**	*Sloanea* sp.	X	
**Erythroxylaceae**	*Erythroxylum anguifugum* Mart.	X	
**Euphorbiaceae**	*Alchornea discolor* Poepp.	X	
	Euphorbiaceae	X	
	*Sapium obovatum* Klotzsch ex Müll. Arg.	X	
**Fabaceae**	*Acacia martiusiana* (Steud.) Burkart		X
	*Albizia inundata* (Mart.) Barneby & J.W. Grimes	X	X
	*Bauhinia* cf. *corniculata* Benth.		X
	*Bauhinia* sp.	X	
	*Cassia grandis* L. f.	X	X
	*Inga vera* Willd.	X	X
	*Lonchocarpus sericeus* (Poir.) Kunth ex DC.		X
	*Mimosa pellita* Humb. & Bonpl. ex Willd.		X
	*Pterocarpus michelii* Britton	X	X
	*Swartzia jorori* Harms	X	X
**Lamiaceae**	*Vitex cymosa* Bertero ex Spreng.	X	X
**Lauraceae**	*Ocotea diospyrifolia* (Meisn.) Mez	X	X
**Myrtaceae**	*Eugenia egensis* DC.	X	X
	*Eugenia polystachya* Rich.	X	
	*Eugenia pseudoverticillata* S. Moore	X	X
	*Eugenia* sp.		X
	*Eugenia* sp. 2		X
	*Eugenia* sp. 3	X	
	*Myrcia splendens* (Sw.) DC.	X	X
**Nyctaginaceae**	*Neea hermaphrodita* S. Moore	X	
**Polygonaceae**	*Coccoloba* cf. *alagoensis* Wedd.	X	X
	*Triplaris americana* L.	X	X
	*Triplaris gardneriana* Wedd.	X	X
**Rubiaceae**	*Genipa americana* L.	X	X
	*Randia armata* (Sw.) DC.	X	X
**Salicaceae**	*Casearia aculeata* Jacq.	X	X
**Sapindaceae**	*Cupania castaneifolia* Mart.	X	X
**Sapotaceae**	*Pouteria glomerata* (Miq.) Radlk.	X	X
**Urticaceae**	*Cecropia pachystachya* Trécul	X	

**Table 2:** Results for the three GLMM models. Interactions were found to be significant for richness and abundance but not for basal area. The applied distribution type is listed beneath the name of the respective dependent variable. Numbers in brackets denote standard errors. Pseudo-R² calculation followed Nakagawa & Schielzeth [40]. The marginal R^2^ shows the variation explained only by the fixed effects while the conditional R^2^ shows the variation of the fixed and random effects. For the negative-binomial abundance model calculation of R² was not possible hence we used the R^2^ of a Poisson model as a proxy.

	*Dependent variable*:		
	Richness	Abundance	Basal area [cm²]
	*Poisson*	*Negative Binomial*	*Gaussian*
	(1)	(2)	(3)
**Intercept**	-3.02** (1.24)	-3.38** (1.57)	-184.09* (111.85)
**Water height**	0.97*** (0.27)	1.31*** (0.35)	86.64*** (24.88)
**Fire**	4.44** (2.13)	5.28** (2.59)	79.22 (166.96)
**Water height** * **Fire**	-1.07** (0.50)	-1.37** (0.60)	-39.49 (38.66)
**Observations (n)**	150	150	150
**R² marginal**	0.16	0.34^+^	0.61
**R² conditional**	0.18	0.40^+^	0.70

*p<0.1

**p<0.05

***p<0.01

+ R² calculated from Poisson GLMM

### Abundance

The total number of individuals recorded was 1,423 trees, shrubs, and palms (1,897 ind. ha^-1^). In burned areas, the total abundance was 1,032 (2,907 ind. ha^-1^) with a mean of 13.06 (SD: 8.6) individuals per plot, while in unburned areas, the total abundance was 391 (990 ind. ha^-1^) with a mean of 5.51 (SD: 4.52) individuals per plot.

The negative binomial GLMM for abundance showed a positive relationship with elevation in burned areas, also having a significant interaction (Fig 4B). There was no effect for the unburned areas, which show constant abundance values of about five individuals per plot (Table 2). Hence, the shorter the time of inundation in burned areas, the more individuals were found.

### Basal area

The total basal area was 3497.8 cm^2^/ha (mean 76.09 cm²; SD: 44.84; min: 9.55 cm²/plot; max: 197.53 cm²/plot) for the burned areas and 6365.6 cm²/ha (mean 49.27 cm²; SD: 40.33; min: 1.91 cm²/plot, max: 168.93 cm²/plot) for unburned areas. In the Gaussian-GLMM, variation on mean basal area per plot was positively associated with topographic position (Table 2), indicating that areas with shorter inundation time have higher sums of basal areas (R^2^ marginal = 0.61 and R^2^ conditional = 0.70). However, variation in basal area was not related to the fire historic and interaction between fire and topographic position (Table 2, Fig 4C).

### Analysis of Similarity and Indicator Species Analysis

**Fig 5:**
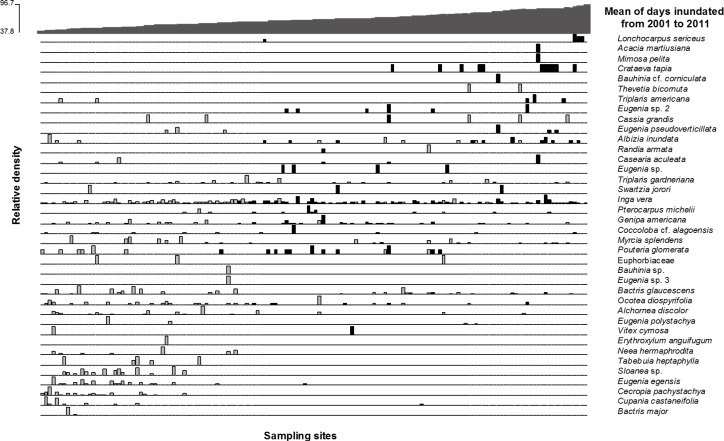
Direct ordination of burned and unburned plots in the riparian forest of Paraguay River, showing the yearly mean time of inundation. Grey bars correspond to burned sites and black bars to unburned sites.

The results of the ANOSIM analysis shows that there are no clear groups formed by the factor groups analyzed (ANOSIM-R = 0.11, 0.07, and 0.13for Unburned/burned, Inundation low/high and the combination of fire and inundation respectively). These results were significant at the 99% significance level after 999 permutations. This demonstrates a strong overlap in species composition. On the other hand the indicator species analysis revealed that 15 out of 39 species were indicative for one of the possible factor combinations.*i*.*e*. occurred significantly more often in only one combination of fire and inundation (Table 3). We found that six species, including *C*. *pachystachya* and *Eugenia egensis*, occurred mainly in burned places, and at a shorter time of inundation. The species *Cassia grandis*, *Myrcia splendens* and *Thevetia bicornuta* indicated burned areas with a longer inundation time. In unburned areas with long time inundation time, *Crataeva tapia* was the most indicative species. *Alchornea discolor* and *Bactris glaucescens* were indicative for burned areas but were indifferent to inundation. *Ocotea diospyrifolia* was indicative of almost all environments except unburned with low levels of inundation. Generally, more species were indicative for burned areas than for unburned areas (Table 3; Fig 5). Other species including *Pouteria glomerata* and *I*. *vera* were found across the whole gradient in burned and unburned places (Fig 5). The main functional trait found in the floodable areas with fire historic was resprouting ability. From the 12 species found as indicator of burned areas, 8 had resprouting ability, one is fast growing, and three have thick bark (Table 3).

**Table 3:** Indicator species analysis for all combinations of the fire regime and two inundation categories. Of the 24 combinations, 5 had species with significant group equalized indicator values (IndVal_g_), and 13 of the 39 tree and shrub species were indicative for a factor combination. Low inundation refers to areas of 4.32–4.85 m, while high inundation refers to areas of 3.79–4.32 m in relation to the zero of the Ladário gauge.

Fire regime / Inundation	Species	IndVal.g	p-value	Func. Traits
**Burned / low inundation**	*Cecropia pachystachya*	0.562	0.001 ***	FG
	*Eugenia egensis*	0.523	0.002 **	RE
	*Sloanea sp*.	0.496	0.002 **	RE
	*Neea hermaphrodita*	0.35	0.025 *	RE
	*Cupania castaneifolia*	0.342	0.037 *	RE
	*Handroanthus heptaphyllus*	0.296	0.05 *	TB
**Burned / high inundation**	*Myrcia splendens*	0.422	0.043 *	RE
	*Cassia grandis*	0.313	0.049 *	TB
	*Thevetia bicornuta*	0.302	0.030 *	RE
**Unburned / low inundation**	*Pouteria glomerata*	0.426	0.023 *	**—**
	*Pterocarpus michelii*	0.383	0.032 *	**—**
**Unburned / high inundation**	*Crataeva tapia*	0.456	0.001 ***	RE
**Burned / low + high inundation**	*Bactris glaucescens*	0.575	0.009 **	RE
	*Alchornea discolor*	0.464	0.007 **	RE
**Burned / low + high inundation & unburned / high inundation**	*Ocotea*.*diospyrifolia*	0.492	0.013 *	TB

Asterisks indicate p-value

* p ≤ 0.05

** p ≤ 0.01

*** p ≤ 0.001.

**(Func. Traits–Additional information on functional traits for fire and/or inundation.)** FG–Fast growing from seeds; RE–High number of branches and high resprouting ability; TB–Thick bark.

In spite of both burned and unburned areas having the same range of low line topographic positions (90 d/yr of inundation), only in the unburned areas we had individuals in the most flooded positions (Fig 5). In the burned areas these topographic positions were occupied mainly by floodable grasslands, which do not appear in Fig 5.

### Species response curves along the inundation gradient

**Fig 6:**
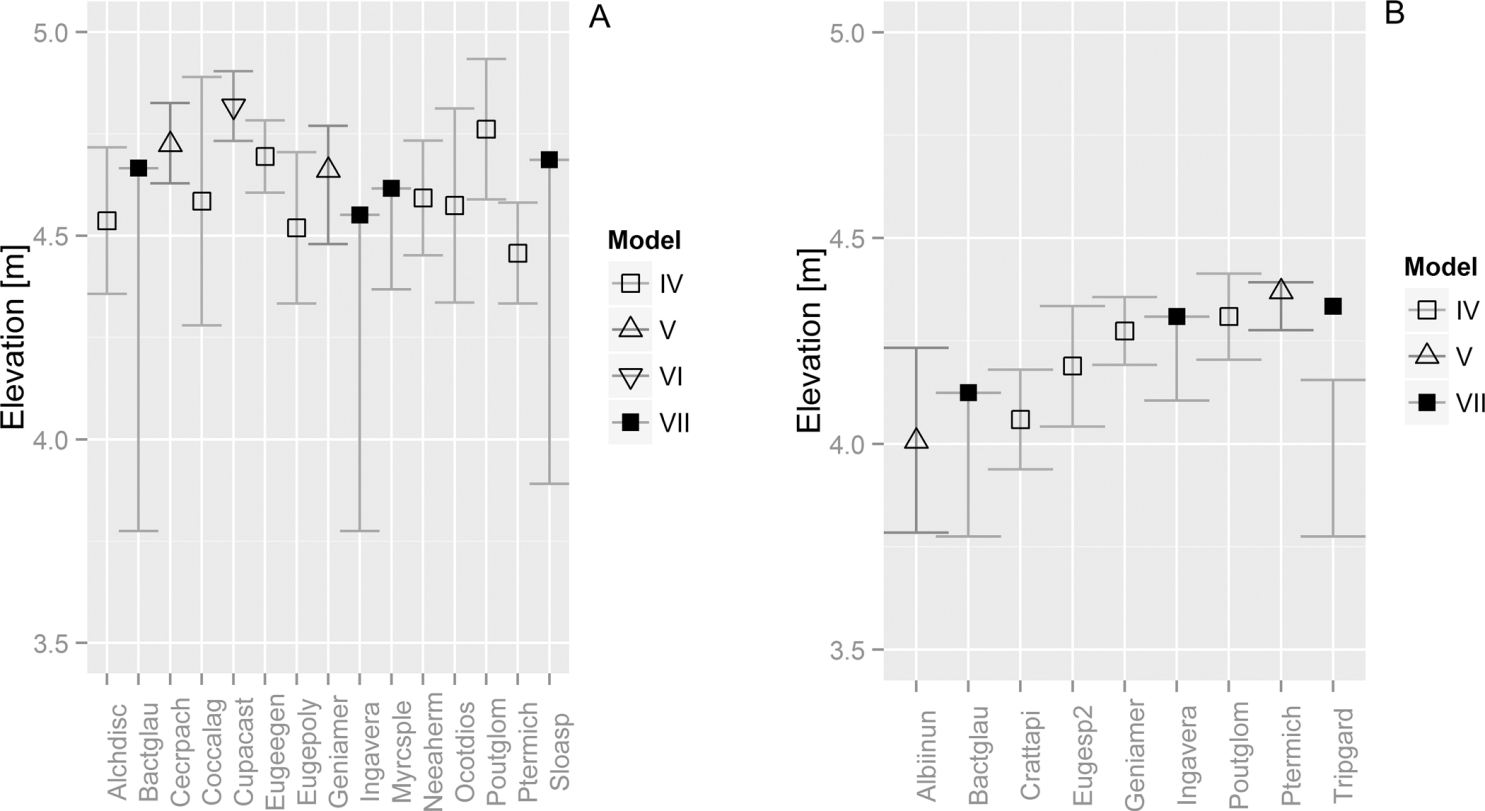
**Species responses to the elevation gradient for (a) burned and (b) unburned areas.** Optima of the specific model types are depicted as symbols, while error bars indicate the tolerance range per species. Response model type IV (unimodal); V (unimodal left skewed); VI (unimodal right skewed) and VII (multimodal). Species names were abbreviated to eight letter codes. Elevation is relative to the Ladário gauge.

In the unburned areas, only nine species showed responses of type IV (4), V (2), and VII (3). Most species had their optima in the higher parts of their flooding gradient occurrence belt, and four had their optima in the middle. Mainly multimodal model VII species (*T*. *gardneriana* and *B*. *glaucescens*) and unimodal left-skewed model V species (*A*. *inundata*) occurred also in the lower parts of the gradient (Fig 6). In the burned areas, 15 species showed responses of type IV (8), V (2), VI (1), and VII (4). Half of the species had their optima in the middle of their flooding gradient occurrence belt, six had their optima in the higher parts, and none had its optimum located in the lower parts. In general, the optima of the species in burned areas were higher than those in the unburned areas (Fig 6). Three species (*Bactris glaucescens*, *Inga vera*, and *Sloanea* sp.), however, showed multimodal responses across almost the whole gradient, with their optima (*i*.*e*. the highest abundance) lying in the upper half of the gradient. Five species showed responses in burned and unburned areas. From these five, *Pouteria glomerata* and *Genipa americana* changed their range of occurrence in burned areas to a higher position, and *G*. *americana* also changed the type of response from IV to V. *B*. *glaucescens* and *I*. *vera* showed the same response (VII) but enlarged their range across the gradient. In burned areas, *Pterocarpus michelli* occurred in the same elevational range but changed its type of response from V to IV (Fig 6).

## Discussion

Our investigations on variations in plant community properties along inundation gradient and occurrence of fire revealed four major findings: (1) as expected, basal area is positively related with reduced inundation time; (2) richness and abundance were also positively related with reduced inundation time but only in burned areas (3) there were 15 species which can be considered as indicators for at least one combination of the studied factors (inundation time, fire or their interaction); (4) nine species in burned area and 15 species in unburned area showed uni- or multimodal response curves along the inundation gradient and from these, five species changed their range of occurrence in burned areas to a higher position, and one also changed the type of response. Furthermore, optima across the elevation gradient were on average approximately 1m higher for the burned areas indicating a shift of the vegetation boundary in burned areas.

### Structural parameters

We found that tree species richness showed a positive relationship with fire in combination with the effect of the water level, according to the duration of inundation. The number of woody species can increase depending on the length of time after a fire event [62], but in Paraguay River system, inundation prevents the occupation of the low lying areas by woody species. Only species that are very resistant to inundation can establish in lower places after a fire. In these areas, the inundation is very predictable and long lasting and can prevent some fast-growing species from establishing. The inundation can reduce the pool of species that can reach an area, mainly because of the restriction caused by oxygen deprivation for the root system and darkness to the leaves of seedlings when they are trying to become established [4, 6, 9, 63–64]. If these features act together frequently, the result is that fire maintains the differences in richness along the inundation gradient. On the other hand, if fire occurs with the same frequency in a set of years without inundation, the expected scenario would be an increase in the number of species due to opening gaps and the encroachment from the neighboring vegetation not subjected to inundation. Sometimes a dry decade occurs, such as in the 1960s when the Paraguay River did not attain the critical level of 4 m for 10 years. In this case, the riparian vegetation is not flooded for this period of time. This interaction is less clear for species richness in the regeneration phase (see Oliveira et al. [33]) probably because the action of inundation can be fully manifested after some time and also after some level of recruitment.

Abundance was higher in burned areas, but it remained the same in unburned areas along the inundation gradient. In another study regarding regeneration under fire and flood influence on forest of the Paraguay River the same tendency was found for abundance of species in the regeneration phase [33]. Burning seems to eliminate some individuals and might open gaps for recruitment of numerous new plants. Nevertheless, the higher abundance is restricted in low-lying areas. As these low-lying areas have neighboring grasslands and fire nearly always comes from there to the forest, the fire would be expected to kill young trees in the lower areas. These young individuals are eliminated when they did not reach the critical size to prevent top killing [65, 66]. This phenomenon is particularly true in savannas [67]. The inundation also acts as a restrictor for abundance, because it is expected that most species that come from higher areas need a period without inundation to germinate and become established [9, 68]. The inundation in the lower areas of the Paraguay River levee can reach over 150 d/yr, hence it decreases the likelihood for a given plant to establish there after a fire event.

According to our expectations, the basal area diminished with increasing flooding duration. The amount of biomass, which can be stored in a variation from 40 to 100 d/yr of inundation, was progressively lower in burned and unburned areas of this community. Increased time inundated can reduce the photosynthetic activity of the species under waterlogged conditions [9, 69], affect water and nutrient uptake [2, 3] and diminish the biomass on low-lying areas [8]. Even though all surveyed plots are flood prone, in areas of about 1 m lower topography the inundation time can be three times longer than on the crest of the levee and can alter plant metabolism. On the other hand, fire was not associated with any changes in this pattern. The response for basal area did not change in terms of accumulated wood along the inundation gradient independently of whether the basal area is distributed in one big stem or in a multistem. This means that fire can affect abundance along the inundation gradient, but the restriction caused by inundation on basal area variation is proportional in burned and unburned areas.

### Species composition

Most of the main species are the same in burned and unburned areas. Species such as *Inga vera* and *Triplaris gardneriana* are resistant to fire and occur with a high number of individuals in both burned and unburned areas. This is why the ANOSIM was not able to detect differences between burned and unburned places. We considered this as resilience in terms of fire occurrence. Nevertheless, when we analyze different combinations of fire occurrence and flood duration, the differences come to light, mainly in the less common species. The indicator species analysis, revealed that some species such as *Eugenia egensis* and *Neea hermaphrodita* were significantly related to higher burned areas (low inundation). These areas were flooded only in 5 of the 10 years analyzed, with a maximum mean of 78 d/yr of inundation which is relatively low. Considering that some of the riparian woody species need a flood free period to grow and establish [9, 68], these areas, after fire, favor fast growing traits such as presented by *Cecropia pachystachya*. In riparian areas along the Paraguay River, it is difficult to distinguish them based on their fire history and to find signs of old burning. Nevertheless, this is the only fast growing species found to be a good indicator for burned areas in these riparian forests, facilitating our understanding of the fire history in this region. *C*. *pachystachya* species is a pioneer and can germinate best under light, occupying vegetation gaps, but may also germinate under shade [70]. It also grows on floating histosols after fire in monodominant stands of *Cyperus giganteus* [71, 72]. Hence, the fast growth is a trait to escape from fire once its size overcomes certain threshold to avoid top killing and to occupy fire-prone and seasonally flooded habitats.

### Functional traits

From the functional traits listed for the identified indicator species, the most noteworthy was resprouting ability, present in almost all species found in burned areas both in lower and higher topographic positions. This functional trait permits them to persist in naturally disturbed environments and speeds up the environmental reconfiguration after a fire event [73, 74]. Resprouting is also a multi-task adaptation for these environments once is not only a response to fire [28], but also to flooding and waterlogging [8, 73]. Some species here, such as *Triplaris gardneriana* and *Myrcia splendens*, can exhibit the same response for both opposite events and this seems to be a key adaptation in maintaining them among the dominants in these environments. This provides the fast recovering of the forest with these species that occupy the persistence niche (*sensu* Bond & Midgely [73]). However, for some species such as *Crataeva tapia*, which has gemmiferous roots, this trait seems not to work for fire as *C*. *tapia* occurred only with low number of individuals in burned areas. One possible explanation is that fire can destroy the ability of this species to resprout, but this must be better investigated.

### Species responses along inundation gradient

The levees of the Paraguay River are occupied by species related to the level and time (duration) of inundation [5]. However, fire changes the ability of some of them to occupy the levee. These species have their preferred environmental optima shifted to a higher position than they would tend to occupy in the absence of fire. Fire pushes the woody species and those adapted to inundation to higher positions on the riverbank. The consequence of these changes is the impoverishment of the lower places in terms of richness and/or abundance. Also, the lower limit of the woody vegetation is higher in burned areas, probably because of the elimination of trees by fire. As fire approaches from the neighboring grasslands, it is reasonable to expect the strongest effect in the transition zone, *i*.*e*. the grassland/forest boundary. In dry years, during the dry season these grasslands are very fire prone. We do not have data about fire intensity; however, we assume that in the ecotone the grasses provide fuel that increases flames and the probability of top killing of trees. This simultaneously controls the encroachment of tree species in open vegetation [65]. This is particularly deleterious for species without the ability to resprout after fire events. One interpretation of these data is that the range of some species can pulse over time. In very dry years with fire occurrence, many woody species shift their optima, i.e. the highest density of individuals, to higher positions. On the other hand, we expect in a highly inundated sequence of years these species can shift their optima to lower areas and are able to grow in areas nowadays occupied by grasses.

For some species such as *Bactris glaucescens* and *Inga vera*, fire appears to enlarge their realized niche along the inundation gradient. Both showed a multimodal response related to their competitiveness. *B*. *glaucescens* is a clumped palm with rhizomes that allow fast resprouting after fire. For *I*. *vera*, this response requires further investigation, but it recruits better in dry years and once established it can be resistant to inundation and fire events. It is a successional species [75], able to grow best under full sunlight, as a pioneer. These features offer insight into why this species is one of the most successful in the riparian forests of the Pantanal [5, 76].

Based on the analysis of the species response curves and in the diminishing effect in richness and abundances in lower places, we hypothesize that fire, along an inundation gradient, can promote shifts in the species optima by imposing a retreat from the lower areas of the inundation gradient and by opening space for a positive response in the upper parts less subjected to inundation stress. This interaction seems to shape the boundary between riparian forest and floodable grasslands in the low-lying areas. Species responses to fire thus require further investigation in wetland ecosystems as well as other regions. Overall, the interaction of fire and time of inundation, when it occurs, seems to be important in some aspects of the structuration of this riparian forest and shows that this interaction deserves more investigation in other flood pulsing environments.

## References

[1] Junk WJ, Bayley PB, Sparks RE. The flood pulse concept in river-floodplain systems. Can Spec Publ Fish Aquat Sci. 1989; 106: 110–127.

[2] Armstrong W, Brandle R, Jackson MB. Mechanisms of flood tolerance in plants. Acta Bot Neerl. 1994; 43: 307–358.

[3] Sairam RK, Kumutha D, Ezhilmathi K, Deshmukh PS, Srivastava GC. Physiology and biochemistry of waterlogging tolerance in plants. Biol Plantarum 2008; 52: 401–412,

[4] Ferreira LV, Stohlgren TJ. Effects of river level fluctuation on plant species richness, diversity and distribution in a floodplain forest in Central Amazonia. Oecologia 1999; 120: 582–587. 10.1007/s004420050893 28308309

[5] Damasceno-Junior GA, Semir J, Santos FAM, Leitão-Filho HF. Structure, distribution of species and inundation in a riparian forest of Rio Paraguai, Pantanal, Brazil. Flora 2005; 200: 119–135.

[6] Wittmann F, Schöngart J, Montero JC, Motzer T, Junk WJ, Piedade MTF, et al Tree species composition and diversity gradients in white-water forests across the Amazon basin. J Biogeogr. 2006; 33: 1334–1347.

[7] Mitsch WJ, Taylor JR, Benson KB. Estimating primary productivity of forested wetland communities in different hydrologic landscapes. Landscape Ecol. 1991; 5, 75–92.

[8] Rodríguez-González PM, Stella JC, Campelo F, Ferreira MT, Albuquerque A. Subsidy or stress? Tree structure and growth in wetland forests along a hydrological gradient in Southern Europe. Forest Ecol Manag. 2010; 259:2015–2025.

[9] Parolin P. Submerged in darkness: adaptations to prolonged submergence by woody species of the Amazonian Floodplains. Ann Botany 2009; 103: 359–376. 19001429 10.1093/aob/mcn216 PMC2707320

[10] Kubitzki K, Ziburski A. Seed dispersal in flood plain forests of Amazonia. Biotropica 1994; 26: 30–43.

[11] Pott A, Pott VJ. Plantas do Pantanal. Brasilia: EMBRAPA-SPI; 1994.

[12] Lockwood JL, Ross MS, Sah JP. Smoke on the Water: The Interplay of Fire and Water Flow on Everglades Restoration. Front Ecol Environ. 2003; 1: 462–468.

[13] Heinl M, Frost P, Vanderpost C, Sliva J. Fire activity on drylands and floodplains in the southern Okavango Delta, Botswana. J Arid Environ. 2007; 68: 77–87.

[14] Macedo HA, Silva A, Neves SMAS, Neves RJ. Avaliação das queimadas no Pantanal do Paraguai na região de Corumbá e Ladário, MS no período de maio de 2009. Anais 2° Simpósio de Geotecnologias no Pantanal, Corumbá, 7–11 novembro 2009, Embrapa Informática Agropecuária/INPE. 2009; p.452-459. Avalilable: http://queimadas.cptec.inpe.br/~rqueimadas/material3os/queima_pantanal_hudsonazevedo.pdf

[15] Stellmes M, Frantz D, Finckh M, Revermann R. Fire frequency, fire seasonality and fire intensity within the Okavango region derived from MODIS fire products. Biodiversity Ecol. 2013; 5: 351–362.

[16] Strohbach BJ. Vegetation of the Okavango river valley in Kavango West, Namibia. In: Oldeland J, Erb C, Finckh M, Jürgens N, editors. Environmental Assessments in the Okavango Region. Biodiversity & Ecology 2013, 5: 321–339.

[17] Gignoux J, Clobert J, Menaut JC. Alternative fire resistance strategies in savanna trees. Oecologia 1997; 110: 576–583 10.1007/s004420050198 28307253

[18] Lawes MJ, Midgley JJ, Clarke PJ. Costs and benefits of relative bark thickness in relation to fire damage: a savanna/forest contrast. J Ecol. 2013; 101: 517–524.

[19] Deng C, Pan X, Zhang H, Pan X. Fire-resistance of six tree species to fire probed by chlorophyll fluorescence. J Food Agric Environ. 2012; 10: 1329–1333.

[20] Lukac M, Pensa M, Schiller G. Tree Species’ Tolerance to Water Stress, Salinity and Fire In: Bredemeier M, Cohen S, Godbold DL, Lode E, Pichler V, Schleppi P, editors. Forest Management and the Water Cycle: An Ecosystem-Based Approach. Ecological Studies 212 New York: Springer Science+Business Media B.V.; 2011 p247–261.

[21] Heinl M, Sliva J, Tacheba B, Murray-Hudson M. The relevance of fire frequency for the floodplain vegetation of the Okavango Delta, Botswana. Afr J Ecol. 2007; 46: 350–358.

[22] Agge JK, Wright CS, Williamsom N, Huff MH. Foliar moisture content of Pacific northwest vegetation and its relation to wildland fire behavior. For Ecol Manage. 2002; 167: 57–66.

[23] Gignoux J, Lahoreau G, Julliard R, Barot S. Establishment and early persistence of tree seedlings in an annually burned savanna. J Ecol. 2009; 97: 484–495.

[24] Stromberg JC, Rychener TJ, Dixon MD. Return of Fire to a Free-Flowing Desert River: Effects on Vegetation. Restor Ecol. 2009; 17: 327–338.

[25] Pettit NE, Naiman RJ. Postfire response of flooding-regenerating riparian vegetation in a semi-arid landscape. Ecology 2007; 88: 2094–2104. 17824440 10.1890/06-1270.1

[26] Bendix J, Cowell CM. Fire, Floods and woody debris: Interactions between biotic and geomorphic processes. Geomorphology 2010; 116: 297–304.

[27] Pettit NE, Naiman RJ. Fire in the riparian zone: Characteristics and Ecological consequences. Ecoystems 2007; 10: 673–687.

[28] Bendix J, Cowell CM. Impacts of Wildfire on the Composition and Structure of Riparian Forests in Southern California. Ecosystems 2010; 13: 99–107.

[29] Joly CA. Flooding tolerance: a reinterpretation of Crawford's metabolic theory. Proc R Soc Edinburgh 1994; 102: 343–354.

[30] Parolin P, Wittmann F, Ferreira LV. Fruit and seed dispersal in Amazonian floodplain trees–a review. Ecotropica 2013; 19: 15–32.

[31] Ab’Saber NA. O Pantanal Mato-Grossense e a teoria dos refúgios. R Bras Geogr. 1988; 50: 9–57.

[32] Silva JSV, Abdon MM. Delimitação do Pantanal Brasileiro e suas sub-regiões. Pesq Agropec Bras. 1998; 33: 1703–1701.

[33] Oliveira MT, Damasceno-Junior GA, Pott A, Paranhos-Filho AC, Suarez YR, Parolin P. Regeneration of riparian forests of the Brazilian Pantanal under flood and fire influence. Forest Ecol Manag. 2014; 331: 256–263.

[34] IBGE. Manual técnico da vegetação brasileira Rio de Janeiro: Ed. da Fundação Brasileira de Geografia e Estatística; 1992.

[35] Kottek M, Grieser J, Beck C, Rudolf B, Rubel F. World map of the Koppen-Geiger climate classification updated. Meteorol Z. 2006; 15: 259–263.

[36] Soriano BMA. Caracterização climática de Corumbá-MS. Boletim de Pesquisa 11 Corumbá: Embrapa Pantanal; 1997.

[37] Hamilton SK, Sippel SJ, Melack JM. Inundation patterns in the Pantanal wetland of South America determined from passive microwave remote sensing. Arch Hydrobiol. 1996; 137: 1–23.

[38] Galdino S, Clarke RT. Probabilidade de ocorrência de cheia no rio Paraguai, em Ladário, MS—Pantanal Circular Técnica 23 Corumbá: Embrapa Pantanal; 1997.

[39] Bertazzoni EC, Damasceno-Junior GA. Aspectos da biologia e fenologia de *Oryza latifolia Desv*.(Poaceae) no Pantanal sul-mato-grossense. Acta Bot Bras. 2011; 2: 476–486.

[40] Fernandes FA, Fernandes AHBM, Soares MTS, Pellegrin LA, Lima IBT. Atualização 491 do mapa de solos da planície pantaneira para o Sistema Brasileiro de Classificação de Solos Comunicado Técnico 61 Corumbá: Embrapa Pantanal; 2007.

[41] Gelman A, Hill J. Data analysis using regression and hierarchical/ multilevel models Cambridge: Cambridge University Press, 2007.

[42] Nakagawa S, Schielzeth H. A general and simple method for obtaining *R*^2^ from generalized linear mixed-effects models. Methods Ecol Evol. 2013; 4: 133–142.

[43] Zuur A, Ieno EN, Walker N, Saveliev AA, Smith GM. Mixed effects models and extensions in ecology with R New York: Springer Science & Business Media, 2009.

[44] Clarke KR. Non-parametric multivariate analyses of changes in community structure. Aust J Ecol. 1993; 18:117–143.

[45] Dufrêne M, Legendre P. Species assemblages and indicator species: The need for a flexible asymetrical approach. Ecol Monogr. 1997; 67:345–366.

[46] De Cáceres M, Legendre P, Moretti M. Improving indicator species analysis by combining groups of sites. Oikos 2010; 119: 1674–1684.

[47] Jansen F, Oksanen J. How to model species responses along ecological gradients—Huisman-Olff-Fresco models revisited. J Veg Sci. 2013; 24: 1108–1117.

[48] Huisman J, Olff H, Fresco LFM. A hierarchical set of models for species response analysis. J Veg Sci. 1993; 4: 37–46.

[49] Oksanen J, Minchin PR. Continuum theory revisited: what shape are species responses along ecological gradients? Ecol Model. 2002; 157: 119–129.

[50] Uğurlu E, Oldeland J. Species response curves of oak species along climatic gradients in Turkey. Int J Biometeorol. 2012; 56: 85–93. doi: 10.1007/s00484-010-0399-9 21249505 10.1007/s00484-010-0399-9

[51] Burnham KP, Anderson DR. Model selection and multimodel inference: a practical information-theoretic approach Second edition, New York: Springer; 2002.

[52] R Development Core Team R: A language and environment for statistical computing R Foundation for Statistical Computing Vienna Austria: 2015; Available: http://www.R-project.org/.

[53] Bates D, Maecher M, Bolker B, Walker S, Christensen RHB, Singmann H, et al. lme4: Linear mixed-effects models using Eigen and S4. R package, version 1.1–10. 2015; Available: https://cran.r-project.org/web/packages/lme4/index.html

[54] Barton K. MuMln: multi-model inference. R package, version 1.15.1. 2015; Available: https://cran.r-project.org/web/packages/MuMIn/MuMIn.pdf

[55] Roberts DW. labdsv: Ordination and Multivariate Analysis for Ecology. R package version 1.7–0. 2015; Available: https://cran.r-project.org/web/packages/labdsv/index.html.

[56] Oksanen J, Blanchet FG, Kindt R, Legendre P, Minchin PR, O'Hara RB, et al. vegan: Community Ecology Package. R package version 2.3–1. 2015; Available: https://cran.r-project.org/web/packages/vegan/index.html

[57] De Cáceres M, Legendre P. Associations between species and groups of sites: indices and statistical inference. Ecology 2009; 90: 3566–3574. 20120823 10.1890/08-1823.1

[58] Hlavac M. stargazer: LaTeX/HTML code and ASCII text for well-formatted regression and summary statistics tables. R package version 5.2. 2015; Available: https://cran.r-project.org/web/packages/stargazer/stargazer.pdf

[59] Wickham H. ggplot2: elegant graphics for data analysis New York: Springer New York; 2009.

[60] Venables WN, Ripley BD. Modern Applied Statistics with S Fourth Edition New York: Springer, 2002.

[61] Breheny P, Burchett W. visreg: Visualization of regression models. R package version 2.0–5. 2015, Available:http://CRAN.R-project.org/package=visreg.

[62] Bell DT, Koch JM. Post-fire succession in the northern jarrah forest of Western Australia. Aust J Ecol. 1980; 5: 9–14.

[63] Crawford RMM. Whole plant adaptations to fluctuating water tables. Folia Geobot. Phytotx. 1996, 31: 7–24,1996.

[64] Koponen P, Nygren P, Sabatier D, Rousteau A, Saur E. Tree species diversity and forest structure in relation to microtopography in a tropical freshwater swamp forest in French Guiana. Plant Ecol. 2004,173: 17–32.

[65] Hoffmann WA, Geiger EL, Gotsch SG, Rossatto DR, Silva LCR, Lau OL, et al Ecological thresholds at the savanna-forest boundary: how plant traits, resources and fire govern the distribution of tropical biomes. Ecol Lett. 2012; 15: 759–768. doi: 10.1111/j.1461-0248.2012.01789.x 22554474 10.1111/j.1461-0248.2012.01789.x

[66] Silva LCR, Hoffmann WA, Rossato DR, Haridasan M, Franco AC, Horwath WR. Can savannas become forests? A coupled analysis of nutrient stocks and fire thresholds in central Brazil. Plant Soil. 2013; 373: 829–842.

[67] Higgins SI, Bond WJ, Combrink H, Craine JM, February EC, Govender N, et al Which traits determine shifts in the abundance of tree species in a fire-prone savanna? J Ecol. 2012; 100: 1400–1410.

[68] Parolin P. Submergence tolerance vs. escape from submergence: two strategies of seedling establishment in Amazonian floodplains. Environ Exper Bot. 2002; 48: 177–186.

[69] Maurenza D, Marenco RA, Piedade MTF. Efeito da inundação de longa duração sob o crescimento de *Pouteria glomerata* (Sapotaceae), uma arbórea da várzea da Amazônia Central. Acta Amaz. 2009; 39: 519–526.

[70] Valio IFM, Scarpa FM. Germination of seeds of tropical pioneer species under controlled and natural conditions. Revta brasil Bot. 2001; 24: 79–84.

[71] Cunha NL, Delatorre M, Rodrigues RB, Vidotto C, Gonçalves F, Scremin-Dias E, et al Structure of aquatic vegetation of a large lake, western border of the Brazilian Pantanal. Braz J Biol. 2012; 72: 519–531. 22990823 10.1590/s1519-69842012000300015

[72] Rocha M, Santos-Junior CC, Damasceno-Junior GA, Pott VJ, Pott A. 2014. Effect of fire on a monodominant floating mat of *Cyperus giganteus* Vahl in a neotropical wetland. Braz J Biol. 2015; 75:114–24. 25945628 10.1590/1519-6984.08613

[73] Bond WJ, Midgley JJ. Ecology of sprouting in woody plants: the persistence niche. Trends Ecol Evol. 2001; 16: 45–51. 11146144 10.1016/s0169-5347(00)02033-4

[74] Clarke PJ, Lawes MJ, Midgley JJ, Lamont BB, Ojeda F, Burrows GE, et al Resprouting as a key functional trait: how buds, protection and resources drive persistence after fire. New Phyt. 2013; 197: 19–35. 10.1111/nph.12001 23110592

[75] Myster RW. Light and nutrient effects on growth and allocation of Inga vera (Leguminosae), a successional tree of Puerto Rico. Can J Forest Res. 2006; 36: 1121–1128.

[76] Wittmann F, Zorzi BT, Tizianel FAT, Urquiza MVS, Faria RR, Sousa NM, et al Tree species composition, structure, and aboveground wood biomass of a riparian forest of the Lower Miranda River, Southern Pantanal, Brazil. Folia Geobot. 2008; 43: 397–411.

